# Sperm storage and spermatozoa interaction with epithelial cells in oviduct of Chinese soft-shelled turtle, *Pelodiscus sinensis*

**DOI:** 10.1002/ece3.1575

**Published:** 2015-07-07

**Authors:** Shaofan Chen, Linli Zhang, Yuan Le, Yasir Waqas, Wei Chen, Qian Zhang, Shakeeb Ullah, Tengfei Liu, Lisi Hu, Quanfu Li, Ping Yang

**Affiliations:** Laboratory of Animal Cell Biology and Embryology, College of Veterinary Medicine, Nanjing Agricultural UniversityNanjing, Jiangsu, 210095, China

**Keywords:** Chinese soft-shelled turtle (*Pelodiscus sinensis*), immune cells, sperm storage, spermatozoa–epithelium interaction

## Abstract

Spermatozoa are known to be stored within the female genital tract after mating in various species to optimize timing of reproductive events such as copulation, fertilization, and ovulation. The mechanism supporting long-term sperm storage is still unclear in turtles. The aim of this study was to investigate the interaction between the spermatozoa and oviduct in Chinese soft-shelled turtle by light and electron microscopy to reveal the potential cytological mechanism of long-term sperm storage. Spermatozoa were stored in isthmus, uterine, and vagina of the oviduct throughout the year, indicating long-term sperm storage in vivo. Sperm heads were always embedded among the cilia and even intercalated into the apical hollowness of the ciliated cells in the oviduct mucosal epithelium. The stored spermatozoa could also gather in the gland conduit. There was no lysosome distribution around the hollowness of the ciliated cell, suggesting that the ciliated cells of the oviduct can support the spermatozoa instead of phagocytosing them in the oviduct. Immune cells were sparse in the epithelium and lamina propria of oviduct, although few were found inside the blood vessel of mucosa, which may be an indication of immune tolerance during sperm storage in the oviduct of the soft-shelled turtle. These characteristics developed in the turtle benefited spermatozoa survival for a long time as extraneous cells in the oviduct of this species. These findings would help to improve the understanding of reproductive regularity and develop strategies of species conservation in the turtle. The Chinese soft-shelled turtle may be a potential model for uncovering the mechanism behind the sperm storage phenomenon.

## Introduction

Sperm storage in the female reproductive tract after mating is used by a variety of animals to maintain fertility, particularly in those species with asynchronous copulation and ovulation (Orr and Zuk 2012). Spermatozoa can be stored in the sperm reservoir of the mammalian reproductive tract for several hours to several days, while reptiles can store them for more than 1 year in the oviduct (Ewing 1943; Birkhead and Møller 1993; Whitaker 2006; Murphy et al. 2009; Phillips et al. 2014). In reptiles, the long-term sperm storage (up to months and years) helps to appropriately synchronize copulation, fertilization, and nesting, to ensure the survival of species. Sperm storage may also function within the context of mate choice (Olsson et al. 1997). The study of mechanisms that govern long-term sperm storage is useful not only to improve the understanding of cell transport, targeting, and communication, but also to clarify the forces that shape sexual selection and ecology (Parker 1970; Neubaum and Wolfner 1998). We currently know little about the mechanisms that support such long-term sperm storage, mainly because evidence from such species is either absent or fragmentary (Holt 2011). If we could uncover the mechanisms behind these processes, thereby facilitating the long-term storage of spermatozoa at ambient temperatures, the practical benefits would be enormous (Holt and Lloyd 2010), particularly for sperm conservation in vitro in liquid state.

The oviduct can affect fertilization broadly by regulating sperm adhesion to the epithelium in mammals (Kadirvel et al. 2012). Studies have demonstrated the direct contact between ejaculated spermatozoa and oviduct epithelial cells is needed for prolonging sperm survival and is especially important for stimulating the *de novo* gene expression in the oviductal cells (Fazeli et al. 2004; Yeste et al. 2009). Spermatozoa bind to epithelial cells lining the oviduct in a cell-restricted manner (Pacey et al. 1995a,b; Kervancioglu et al. 2000; Suarez and Pacey 2006), where they are maintained alive until fertilization (Druart 2012). During spermatozoa transit through the female reproductive tract, spermatozoa encounter different environments with various mechanical properties. Identification of the participants in the dialogue between spermatozoa and oviduct in vivo will require further research, and this will be important to properly elucidate the mechanisms by which spermatozoa survive in the female genital tract (Druart 2012). However, information of relationships between spermatozoa and oviduct is sparse in reptiles.

The Chinese soft-shelled turtle *Pelodiscus sinensi*s is an important aquaculture reptile, which is widely raised in freshwater lakes of central and southern China. But the wild population of turtle is threatened from overfishing, habitat loss, and their low fertility (He 2007). Therefore, several turtle nature reserves have been established in China. Some reports have focused on the reproductive biology of this species for their segregation of spermiation, copulation, and ovulation. The spermiogenesis, spermatozoon morphology, and oogenesis have been characterized to some extent (Chen et al. 2006; Zhang et al. 2007; Nainan et al. 2010). However, there is still a limited knowledge on oviductal sperm storage, an important reproductive strategy in the turtle. This study will analyze the interaction between stored spermatozoa and oviduct epithelial cells by structural and ultrastructural evidences, to reveal the morphological mechanism of long-term sperm storage in *P. sinensis,* which would help to improve the understanding of reproductive regularity and contribute to further studies on the sperm conservation in vitro to keep the wild turtle species going.

## Materials and Methods

### Animals

Twenty adult female Chinese soft-shelled turtles, aged 3–4, from a single pond in Nanjing, southeastern China, were used in this study. Animals were anaesthetized by intraperitoneal administration of sodium pentobarbital (20 mg/kg) and killed by cervical dislocation. They were slaughtered in July (summer), November (autumn), January (winter), and April (spring), five turtles at each time. The oviduct and spermatozoa were collected and fixed (details below) immediately for light and electron microscopy, respectively. The sample procedures were conducted in accordance with the Animal Research Institute Committee guidelines of Nanjing Agriculture University. All efforts were made to minimize animal suffering. The protocol was approved by the Science and Technology Agency of Jiangsu Province (SYXK (SU) 2010-0005).

### Light microscopy

The tissues were embedded in paraffin wax after fixed with 10% neutral buffered formalin (v/v) overnight and serially sectioned (at 5 *μ*m). The sections were stained with hematoxylin and eosin procedure (Harry’s hematoxylin for 2 min and 1% eosin for 30 sec) and examined using an Olympus microscope (BX53; Olympus, Tokyo, Japan) (Bian et al. 2013b).

### Transmission electron microscopy (TEM)

The samples were cut into small blocks (1 mm^3^), fixed in a mixture of 2.5% (v/v) glutaraldehyde in phosphate buffered saline (PBS; 4°C, pH 7.4, 0.1 mol/L) for 24 h, and then postfixed for 1 h at room temperature in the same buffered 1% (w/v) osmium tetroxide. After dehydrated in ascending concentrations of ethyl alcohol, samples were infiltrated with a propylene oxide–Araldite mixture and embedded in Araldite. The blocks were sectioned (50 nm). The ultrathin sections were mounted on Formvar-coated grids and stained with uranyl acetate and lead citrate (Reynolds 1963). The sections were examined and photographed with a transmission electron microscope (H-7650; Hitachi, Tokyo, Japan).

### Scanning electron microscopy (SEM)

Spermatozoa collected from the oviduct were concentrated by centrifugation and resuspended in 2.5% (v/v) glutaraldehyde for 3 h. After washing in cacodylate buffer, the samples were postfixed in 1% (w/v) osmium tetroxide for 1 h. The samples were dehydrated through a graded ethanol series. A drop of spermatozoa suspended in ethanol was placed on a glass coverslip, and the spermatozoa were allowed to settle. The samples were taken through critical point drying in CO_2_ and coated with gold (Sanders et al. 1975). Images were taken with scanning electron microscope (S-520; Hitachi, Tokyo, Japan).

## Results

**Figure 1:**
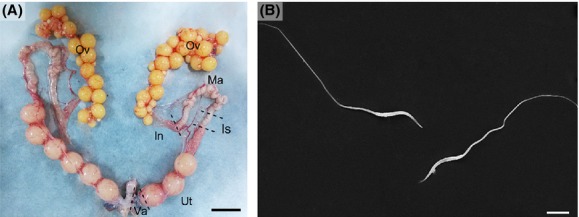
The oviduct and stored spermatozoa of soft-shelled turtle. (A) Photograph of oviduct containing shelled eggs in uterine (Ut). (B) SEM image of the spermatozoa from oviduct. ovary (Ov), infundibulum (In), magnum (Ma), isthmus (Is), uterine (Ut), vagina (Va). Scale bar = 2 cm (A) and 6 *μ*m (B).

**Figure 2:**
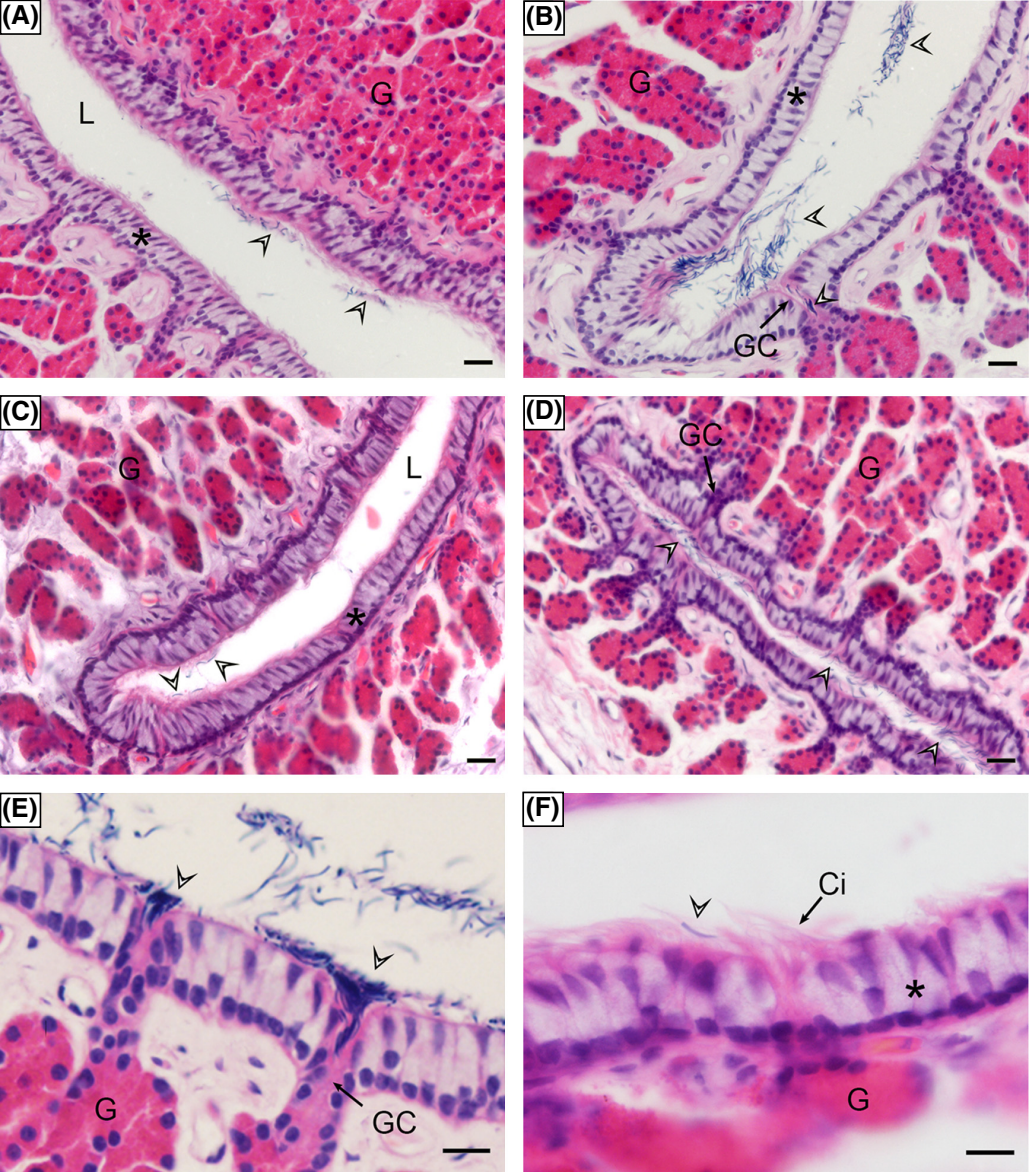
Distribution of spermatozoa through the reproductive tract of soft-shelled turtle during the storage period, H-E stain. Spermatozoa were stored in isthmus, uterine, and vagina of the oviduct. The spermatozoa were either attached to the epithelial surface, embedded among the cilia, gathered in the gland conduit, or moved away from epithelium. (A) Vagina in July. (B) Uterine in November. (C) Isthmus in January. (D) Isthmus in April. (E) Spermatozoa in the gland conduit of isthmus. (F) Spermatozoa were embedded among the cilia of uterine. capillary (↑), cilia (Ci), epithelium (*), gland (G), gland conduit (GC), lumen (L), spermatozoa (arrowhead). Scale bar = 20 *μ*m (A–D) and 10 *μ*m (E, F).

The oviduct of the Chinese soft-shelled turtle was divided into five segments: infundibulum, magnum, isthmus, uterine, and vagina (Fig.1A). After mating from June to August, many spermatozoa (Fig.1B) were observed within the oviduct in July (Fig.2A), November (Fig.2B), the following January (Fig.2C), and April (Fig.2D). These spermatozoa were mainly stored in isthmus (Fig.2C and D), uterine (Fig.2B), and vagina (Fig.2A) of the oviduct in this species.

**Figure 3:**
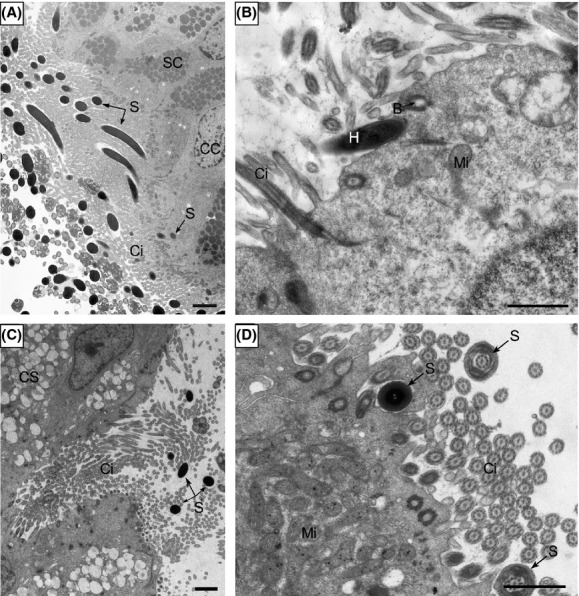
Transmission electron microscopy showing spermatozoal attachment in the oviduct of Chinese soft-shelled turtle. (A) Many spermatozoa (S) were embedded among cilia (Ci) and inserted into the apical hollowness of the ciliated cell (CC). (B) A spermatozoon inserted its head (H) into the hollowness which was surrounded by no lysosome in ciliated cell. (C) Spermatozoa were present at the opening of gland conduit. (D) Cross section of the spermatozoa head in the hollowness of ciliated cell and the spermatozoa midpiece among cilia. basal body (B), secretory cell (SC), mitochondrion (Mi). Scale bar = 2 *μ*m (A, C) and 1 *μ*m (B, D).

Spermatozoa were either attached to the epithelial surface, embedded among the cilia, gathered in the gland conduit, or moved away from epithelium (Fig.2). Heads of many spermatozoa were embedded among the cilia (Fig.2F, 3A and C), and some even intercalated into the hollowness at the apical part of the ciliated cell in the epithelium (Fig.3A, B and D) although many stored spermatozoa were distributed within the oviduct lumen. No lysosomes were distributed around the hollowness of the ciliated cells (Fig.3B and D). The stored spermatozoa often went into the gland conduit of the oviduct (Fig.2B and E).

**Figure 4:**
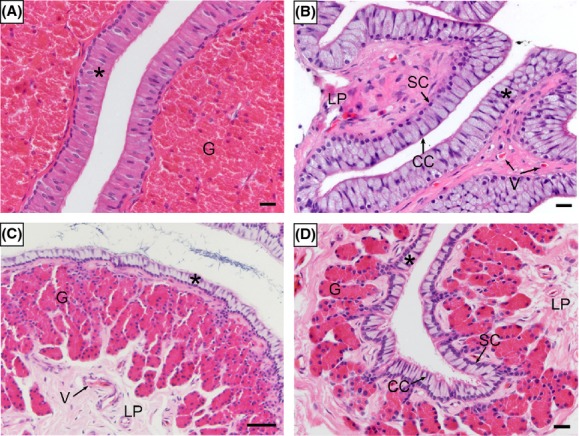
Immune cells were scarce in the mucosa of the oviduct. (A) Magnum. (B) Isthmus. (C) Uterine. (D) Vagina. ciliated cells (CC), epithelium (*), gland (G), lamina propria (LP), secretory cells (SC), blood vessel (V). Scale bar = 50 *μ*m (C) and 10 *μ*m (A, B, D).

**Figure 5:**
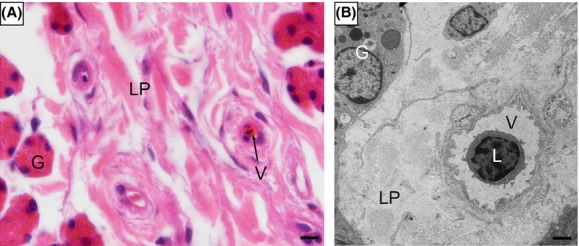
Few immune cells occasionally appeared inside the blood vessel of the lamina propria. (A) The blood vessel in the mucosa of infundibulum, H-E stain. (B) Lymphocyte inside the blood vessel of the lamina propria (LP) in the uterine, TEM. oviduct gland (G), lamina propria (LP), lymphocyte (L), blood vessel (V). Scale bar = 10 *μ*m (A) and 2 *μ*m (B).

Immune cells were sparse in the mucosa of oviduct. It is difficult to detect intraepithelial lymphocyte (IEL), lamina propria lymphocytes (LPLs), plasma cells, dendritic cells, neutrophils, macrophages, and mast cells in the layer of epithelium (Fig.4), except for some lymphocytes inside the blood vessel in lamina propria (Fig.5).

## Discussion

### Spermatozoa can be stored in the oviduct for at least 1 year

Spermatogenesis in seminiferous tubule of the Chinese soft-shelled turtle was a seasonally dependent event (Zhang et al. 2007), and spermiation took place only in October of the later autumn in Nanjing. Immature spermatozoa were then transferred into the epididymis and stored there until the following May (Bian et al. 2013a). After mating during the period from June to August, the spermatozoa were stored in the oviduct, waiting to fertilize eggs. In the present study, lots of spermatozoa were found in the oviduct throughout the year, showing that the sperm storage in this turtle can be maintained for more than 1 year. The sites of sperm storage are not consistent across reptilian species (Gist and Jones 1987; Holt and Lloyd 2010). Sperm storage occurs in both the anterior vagina and infundibulum of oviduct in lizards and snakes (Gist and Jones 1987). In turtles, the studies of Gist and Jones (1989) have established that the spermatozoa are predominantly located at a small region of posterior albumen region (midway between the ovary and vagina) of the oviduct. However, in the present study, spermatozoa were identified in the isthmus, uterine, and vagina of the oviduct. A larger storage area of storage site was also found in the painted turtles and the slider turtles, including the posterior albumen and uterine regions of the oviduct (Gist and Congdon 1998). Compared with those turtle species that store spermatozoa in posterior albumen region, the Chinese soft-shelled turtle may have a greater sperm storage capacity and thus could contain more spermatozoa from multiple males or greater ejaculate masses in a single copulation. It is suggested that fertilization occurs either in the infundibulum or in the albumen regions in reptilian species (Girling 2002). However, we do not know the mechanism by which spermatozoa are released from storage sites, nor whether the oviduct plays a role in the movement of sperm up the oviduct to the site of fertilization. In the oviduct of *P. sinensis*, the broader distribution of spermatozoa indicated diverse abilities of spermatozoa movement after mating. Furthermore, the stored spermatozoa in isthmus, which were closer to the site of fertilization than those spermatozoa in the vagina, would probably reach the eggs more quickly during fertilization. Due to sperm storage for a long time over a broader region, as well as the easy acquisition of this animal, *P. sinensis* may be an effective model for uncovering the mechanisms behind the long-term storage at ambient temperatures.

### Epithelial cells can protect and support spermatozoa in the oviduct

Direct interactions between spermatozoa and oviduct have been reported in diverse species and are common in mammals. However, they have been less reported in reptiles and aves. As far as we are aware, spermatozoa are described as residing free in the lumen of sperm storage sites, without direct connection with the oviductal tissue in reptiles (Kumari et al. 1990; Gist and Fischer 1993; Gist et al. 2008). In the keeled earless lizard, *Holbrookia propinqua*, the spermatozoa were rarely considered to be partially embedded in oviductal tissue (Adams and Cooper 1988). The difference of the sperm–epithelium relationships between reptilian species implies diverse mechanisms of the long-term sperm storage. Interaction between spermatozoa and oviduct can lengthen the life span of spermatozoa, regulate spermatozoa maturation, and affect the fertilizing ability of spermatozoa in mammals (Miller 2011). In vitro, binding to the oviductal epithelium has been shown to prolong the motile life span of spermatozoa in several species (Raychoudhury and Suarez 1991; Pacey et al. 1995b; Apichela et al. 2009). Strategies underlying sperm binding have been considered, especially in terms of preovulatory sperm storage and suppression of full membranous maturation (Hunter 2011). On the other hand, the protective effects of spermatozoon–oviductal epithelial cell interaction against oxidative stress in human spermatozoa have been demonstrated (Huang et al. 2013).

In the present study, direct interactions between spermatozoa and oviduct were found. The sperm heads of the Chinese soft-shelled turtle were always embedded among cilia and even intercalated into the apical hollowness of the ciliated cell in epithelium during storage in the oviduct. There was no lysosome around the apical hollowness, indicating that the ciliated cell can support the spermatozoon instead of phagocytosing in the oviduct. The ciliated cells therefore probably have a role in maintaining sperm storage within the oviduct for a long time in this animal. The protective effects of the spermatozoon–oviductal epithelial cell interaction may enhance the ability of resistance to oxidative stress in turtle spermatozoa, to extend spermatozoon life span during long-term sperm storage. Furthermore, the turtle spermatozoa were often present in the gland conduit of oviduct, where they could easily get the gland secretion for nourishment during sperm storage. The overall effect of oviduct secretions appears to be an increase in spermatozoa life span (Miller 2011).

### Possible immune tolerance in oviduct

There are specific locations in animal tissues and organs where alloantigens and autoantigens are tolerated by the immune system. The genital tract is a unique immunological environment that must both support the reproductive function and resist infection. A compromise state must be established that will allow selective immune privilege for gametes and the developing fetus within the context of an otherwise immunocompetent female reproductive system (Clark and Schust 2013). There is a full set of active immune cells in the female reproductive tract (FRT), and the differential regulation of these cells in the distinct compartments of the FRT is critical for reproductive success (Dunbar et al. 2012). Seminal fluid not only induces the expression of proinflammatory cytokines and chemokines in the cervix, but also causes a major influx of macrophages, DCs, and memory T cells (Sharkey et al. 2012). The stored spermatozoa were supposed to induce the increase of immune cells in the oviduct to remove alloantigen, whereas fewer immune cells were found in the mucosa of the female genital tract in *P. sinensis*. The suppressed immune level in the oviduct has been found in some species with sperm storage. Research on ants has shown that more males contributing to the stored spermatozoa could reduce female immune response during the stage of sperm storage (Baer et al. 2006). Furthermore, in chickens, immune tolerance was considered to be necessary for storage of antigenic spermatozoon to extend the period of time (Bakst 2011). The sparse immune cells may benefit spermatozoa hidden from the immune attack in the female reproductive tract, which suggests that spermatozoa might be immune-privileged during storage in the oviduct of the Chinese soft-shelled turtle. In other words, sperm storage may induce immune tolerance in female reproductive tract of the Chinese soft-shelled turtle.

In conclusion, this study provides direct evidence that spermatozoa can be stored within isthmus, uterine, and vagina for a long time in the Chinese soft-shelled turtle after natural mating. The protection and/or nutrition of the oviduct epithelium, and the small population of immune cells in the genital tract would facilitate long-term sperm storage. In addition, Chinese soft-shelled turtle may be a potential model for uncovering the mechanism behind the sperm storage phenomenon.
